# Mitosis detection in histopathological images using customized deep learning and hybrid optimization algorithms

**DOI:** 10.1371/journal.pone.0327567

**Published:** 2025-07-10

**Authors:** Afnan M. Alhassan, Nouf I. Altmami

**Affiliations:** Department of Computer Science, College of Computing and Information Technology, Shaqra University, Shaqra, Saudi Arabia; University of Vermont, UNITED STATES OF AMERICA

## Abstract

Identifying mitosis is crucial for cancer diagnosis, but accurate detection remains difficult because of class imbalance and complex morphological variations in histopathological images. To overcome this challenge, we propose a Customized Deep Learning (CDL) model, which integrates advanced deep-learning techniques for better mitosis detection. The CDL model utilizes transfer learning to counter the effects of class imbalance and speed up convergence, while skip connections are also employed to improve the localization of mitosis. Furthermore, we have established an innovative selection mechanism by the hybrid of Jellyfish Search Optimizer (JSO) and Walrus Optimization Algorithm (WOA) to maximize the momentum of the model. The proposed approach is rigorously evaluated on multiple publicly available mitosis detection datasets, including Mitosis WSI CCMCT Training Set, Mitosis-AIC, Mitosis Detection, and Mitosis and Non-Mitosis datasets. To tackle these issues, we hereby bring forth a specifically tailored Custom Deep Learning model, that assimilates hybrid CNN architecture into transfer learning and feature selection for improved mitotic detection. The CDL model comprises a Transfer Learning-based Mitosis Detection module under which extracted features from pre-trained deep networks are used to bolster feature extraction and alleviate class imbalance through skip connections to better localize mitosis. The robust assessment on a benchmark dataset displays the outstanding efficacy of the CDL model, reaching an excellent F1 score of 0.994 and accuracy of 98.8% thus proving its strength for the detection of mitotic figures. This proposed methodology can greatly empower pathologists for accurate appraisal of cancer diagnosis and prognosis. Future lines of exploration will include fusion methodologies and time efficiency for real-time applications, as well as extending CDL to various histopathological analyses.

## 1. Introduction

Cancer remains a formidable global health challenge, claiming millions of lives annually and necessitating continuous advancements in diagnostic techniques [1]. Among the various forms of cancer, breast cancer ranks as the most widespread cancer among women worldwide. Its impact is profound, leading to high incidence and fatality rates. Accurate cancer diagnosis is paramount in determining the appropriate treatment strategy and improving patient outcomes [2]. One of the key factors in assessing cancer aggressiveness is mitotic activity, which refers to the process of cell division leading to tumor growth. Traditionally, pathologists analyze histopathological images to identify mitotic figures, intricate structures that signify cancer progression and metastatic potential [3]. However, this manual process is intricate, time-consuming, and susceptible to human error, especially due to the diverse forms of mitotic stages and the presence of structures resembling mitotic figures.

Mitosis, the process by which a cell divides into two identical daughter cells, is visually complex, encompassing several stages such as prophase, metaphase, anaphase, and telophase [4–6]. Each stage presents distinct shapes and textures, making accurate identification a daunting task. Additionally, apoptotic cells and certain non-mitotic nuclei, appearing similar to mitotic figures, add to the complexity. These challenges, coupled with potential variations in slide preparation techniques, create a risk of misdiagnosis and necessitate a more precise and efficient approach [7].

Recent advancements in artificial intelligence (AI), particularly deep learning techniques, have opened new avenues in medical image analysis. Leveraging the power of deep learning, researchers are developing automated Mitosis Detection Systems (MDS) to enhance the accuracy and efficiency of cancer diagnosis [8,9]. Deep learning models, trained on vast datasets of histopathological images, possess the ability to recognize intricate patterns and structures within these images, including mitotic figures and their various stages.

Early studies in mitosis detection primarily relied on traditional image processing techniques, which often struggled with the intricate and diverse morphological features of mitotic figures. Existing models also face difficulties in balancing sensitivity and specificity, often producing false positives due to the presence of apoptotic cells or other structures resembling mitotic figures. Additionally, many deep learning approaches require extensive computational resources and large annotated datasets, which are not always available in clinical settings. With the emergence of deep learning, researchers began exploring convolutional neural networks (CNNs), and recently, intelligent optimization algorithms have been successfully applied to tune hyperparameters in deep learning models, improving convergence and overall performance to address these challenges [10]. CNNs, specifically designed to recognize patterns in images, have shown promising results in accurately identifying mitotic figures [11]. CNN has become a cornerstone of image-based classification tasks in medical imaging due to its ability to learn hierarchical features from data [12,13].

One significant challenge discussed in the literature pertains to the diversity of mitotic stages. Mitosis comprises distinct phases, each exhibiting unique characteristics. One notable challenge is the variability introduced by different staining protocols, image acquisition devices, and preparation techniques across medical institutions, which can drastically affect image appearance and quality. Recent studies have focused on developing algorithms capable of classifying mitotic figures into specific stages, enhancing the granularity of analysis, and providing valuable insights into cancer progression [14]. Current deep learning models often struggle to generalize effectively across datasets with such variations, leading to inconsistent performance in real-world clinical scenarios. Moreover, differences in pathological tissue types, tumor subtypes, and morphological heterogeneity further complicate accurate detection, as models trained on limited or homogenous datasets may fail to adapt to unseen cases. Ambiguity in histopathological images, caused by structures resembling mitotic figures, presents another hurdle. Researchers have worked on refining deep learning models to differentiate between mitotic and non-mitotic structures, employing techniques such as data augmentation and adversarial training to improve model robustness and reduce false positives [15]. These limitations highlight the necessity of developing more robust, adaptive, and efficient mitosis detection systems capable of maintaining high accuracy across diverse histopathological conditions. Some studies have explored interpretable deep learning models, aiming to elucidate the decision-making processes of these algorithms. Techniques like attention mechanisms and explainable AI have been integrated to enhance the interpretability of mitosis detection systems.

Additionally, the integration of mitosis detection into the broader clinical context has gained attention [16–19]. Researchers have begun exploring methods to combine mitosis detection data with other clinical parameters, such as genetic markers and patient history, providing a comprehensive basis for treatment decisions [20]. This integration enhances the overall diagnostic accuracy and aids in personalized cancer therapies.

While progress has been made, challenges persist, including the need for diverse and representative datasets, real-time application of algorithms, and addressing computational scalability. Bridging these gaps is essential for the successful implementation of automated mitosis detection systems in clinical practice, ultimately leading to more precise cancer diagnoses and improved patient outcomes.

The contributions made in this paper are as follows:

- The pre-processing phase introduces novel stain normalization techniques, tailored to the specific requirements of mitosis detection. This innovation contributes to the consistency of images, minimizing variations and improving the quality of subsequent analyses.
- The Hybrid Feature Selection Algorithm proposed in this research is a synergistic blend of two distinct optimization algorithms: JSO and the WaOA. The algorithms work collaboratively to select features that collectively contribute to the discrimination between mitotic and non-mitotic patterns. By combining the global and local search capabilities of JSO and WaOA, respectively, the Hybrid Feature Selection Algorithm achieves a well-rounded exploration of the feature space. This strategy aims to improve the robustness of feature selection for mitosis detection, ensuring relevant features are retained while irrelevant or redundant ones are discarded.
- The customized CNN architecture, integrated with transfer learning. Transfer learning leverages pre-trained models, such as those trained on large datasets like ImageNet, to enhance the performance of the mitosis detection model. The customization of the CNN architecture indicates a tailored design to suit the intricacies of mitosis detection in pathology images. CDL employs a specific loss function, namely focal loss, to optimize the network’s performance for the task of mitosis detection. Focal loss is designed to address the class imbalance inherent in mitosis detection, where mitotic nuclei are significantly outnumbered by non-mitotic nuclei. This strategic choice contributes to improved model training and performance.

The rest of the manuscript is organized in the following way: A strict discussion on the problems of mitosis detection in histopathological images is carried out under Section 2 by combining all existing deep learning methodologies and limitations. Section 3 describes the Customized Deep Learning (CDL) model which covers the data preprocessing, segmentation, mitosis detection, and hybrid feature selection mechanism using Jellyfish Search Optimizer (JSO) and Walrus Optimization Algorithm (WOA). The experimental setup along with the dataset details and the implementation settings employed in this study, as well as the evaluation metrics, can be found in Section 4. Following that is a comparative analysis between CDL and established deep learning models. Lastly, in Section 5, the entire work is concluded with a summation of most of the findings, potential limitations of the study, and possible recommendations for future works, such as improving real-time applicability and expanding CDL to other histopathological image analysis tasks.

## 2. Related works

Chawan Piansaddhayanaon et al. [19] suggested a three-improvement Refine Cascade Network (ReCasNet), an enhanced DL pipeline that addresses the aforementioned issues. During the detection stage, the quantity of low-quality false positives was reduced by window displacement. Additionally, another DL model was employed for object re-cropping to correct items that weren’t properly centered. Enhanced data selection procedures were implemented during the classification stage to reduce mismatches in training data distributions. The evaluation results showed a 4.8% improvement in mitotic cell identification F1 scores, while the mean absolute error (MAE) for mitotic count prediction decreased by 44.1%. The underlying methods of ReCasNet can be applied to different two-stage object identification pipelines, potentially enhancing DL models in general digital pathology applications.

Xiyue Wang et al. [20] introduced the FMDet algorithm, independently verified on multicenter breast histopathology images, as a generalizable and reliable mitotic identification method. They reframed the object detection task as a semantic segmentation issue to capture more nuanced morphological properties of cells. A robust feature extractor was created in their segmentation framework to capture the differences in the appearance of mitotic cells. This feature extractor was built by fusing a channel-wise multi-scale attention mechanism with a fully convolutional network structure. They utilized a Fourier-based data augmentation technique to swap the low-frequency spectrum across two domains, reducing domain differences. The final results demonstrated the algorithm’s potential for use in clinical practice as an assistant decision support tool.

Hameed Ullah Khan et al. [21] proposed the SMDetector, a DL model designed to detect small objects like mitotic and non-mitotic nuclei. To prevent small objects from vanishing in the deep layers, this model utilized dilated layers in the backbone. Additionally, dilated layers were employed to close the size gap between the image and the objects it contains. The region proposal network was designed for precise small object identification. The suggested model achieved an f-measure of 63.88% and an average precision for mitotic nuclei of 68.49%, along with a recall for mitotic nuclei of 59.86%.

Wafaa Rajaa Drioua et al. [22] proposed two methods for semantic picture segmentation of breast cancer histopathology. They introduced an enhanced U-Net architecture for supervised segmentation and an autoencoder architecture for unsupervised segmentation. These models were evaluated on a public dataset of breast cancer histology pictures using various evaluation indicators to assess their segmentation approaches. The outcomes were comparable to those of other contemporary methods, indicating the effectiveness of their segmentation techniques.

Fuat Akal et al. [23] suggested employing DL-based image processing techniques to identify mitoses in histopathology pictures of neuroendocrine tumors. They incorporated the YOLOv5 transform module, a popular object identification technique, into their approach. The YOLOv5 transformer model recognized mitotic cells with an accuracy of 0.89, a recall of 0.68, and an F1 score of 0.77. It performed with 0.80 accuracy, 0.67 recall, and 0.73 F1 score. This streamlined process enables accurate and swift diagnosis, facilitating efficient determination of the tumor’s grade, treatment, and patient monitoring.

Deshmukh Pramod Bhausaheb et al. [24] introduced the concept of a Shuffled Shepherd Deer Hunting Optimization-based Deep Neural Network (SSDHO-based DNN) for accurate breast cancer classification. This approach categorizes photographs of breast tumors into six different groups, including non-tubule, non-tumor nuclei, tubule, apoptosis, tumor nuclei, and mitosis. The proposed method, known as SSDHO, combines the Shuffled Shepherd Optimization Approach (SSOA) and the Deer Hunting Optimization Algorithm (DHOA). The DNN enhances the effectiveness of the breast cancer classification process and generates precise classification results due to its connected hidden neurons. This approach achieved outstanding results, boasting a remarkable accuracy rate of 95.6%.

Nooshin Nemati et al. [25] developed a novel system for detecting and categorizing mitosis in breast histopathology images. Initially, YOLOv5 was utilized to identify cells with a mitotic form; however, the accuracy and reliability of diagnosis were compromised due to its limitations. To address this, fuzzy-based classifiers like Fuzzy-based K Nearest Neighbour, Fuzzy Min-Max, and Fuzzy Random Forest were employed to distinguish mitotic cells from non-mitotic cells after the detection of mitotic-shaped cells with YOLOv5. The effectiveness of this methodology was evaluated on the MITOS ICPR14 dataset, considering Precision, Recall, and F1-Score metrics.

Abdul Rahim Shihabuddin et al. [26] demonstrated the value of a multi-CNN framework for mitosis detection, utilizing three pre-trained CNNs: VGG16, ResNet50, and DenseNet201. Features were extracted from histopathological data using these pre-trained networks. The proposed framework leveraged all 73 folders from the TUPAC16 dataset and all training folders from the MITOS dataset, made available for the MITOS-ATYPIA contest 2014. The accuracy rates for each pre-trained CNN model, VGG16, ResNet50, and DenseNet201, were 83.22%, 73.67%, and 81.75%, respectively. Various combinations of these pre-trained CNNs were used in a multi-CNN framework. Comparing performance metrics with other classifiers such as Adaboost and Random Forest, multi-CNN combinations with three pre-trained CNNs and Linear SVM achieved impressive results with 93.81% precision and 92.41% F1-score.

B. L. et al. (2024) [27] proposed a deep learning framework improvement, DeepMitosisNet, incorporated with the Teaching-Learning-Based Optimization (TLBO) algorithm in order to improve detection accuracy for mitosis events. The model has been validated on the MITOS-ATYPIA 14 dataset using the Aperio and Hamamatsu scanners. The deep learning model achieved an F-score of 96%, precision of 93.7%, and recall of 98%, thus showing superiority over other state-of-the-art techniques in terms of classification accuracy and computational efficiency.

Gonzalez et al. (2023) [28] elaborated on the effect of rotation invariant strategies for mitosis detection in YOLO-based deep learning models. This included an offline augmentation procedure that mainly focused on rotation operations to mitigate the loss and clipping of mitoses. In the comparison of YOLOv4 and YOLOv5, it was found that YOLOv5 in conjunction with both these offline and online rotation augmentation had the highest F1-score, which confirmed its robustness against different oriented mitosis.

This machine learning approach was applied to the brightfield time-lapse imaging for detecting label-free cancer stem-like cell fate by Chambost et al., 2022 [29]. The deep learning algorithm they developed associated the fates and states of individual cells in time, and outperformed existing models based on traditional computer vision and shallow learning. The accuracy and computational performance of this algorithm were superior to those of pre-trained convolutional neural networks (CNNs), which benefits model customization for specific image-analysis problems.

Çayır et al. (2022) [30] designed and developed to research a novel two-stage deep learning technique, MITNET, for mitosis recognition in whole slide images (WSIs) of breast cancer. Two datasets were designed during this study: one with a total of 139,124 annotated nuclei encoded within a total of 1749 patches, and another with 4908 images of mitotic and non-mitotic cells harvested from images of 214 WSIs. MITNET consists of two architectures: MITNET-det for detecting nuclei, which uses CSPDarknet and Path Aggregation Network (PANet) for feature extraction and fusion and has a scaled-YOLOv4 detection strategy; and MITNET-rec for mitosis classification, which processes isolated nucleus images. This approach led to a considerable improvement in mitosis recognition accuracy and optimized applicable deep learning techniques for whole slide image analysis.

## 3. Proposed CDL-based mitosis detection model

**Fig 1:**
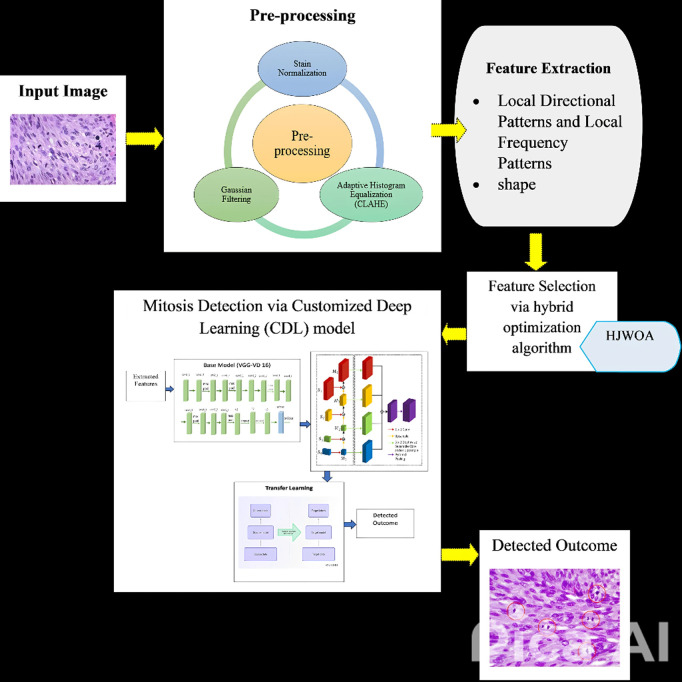
Block diagram of the proposed mitosis detection technique.

The proposed CDL model is a comprehensive framework designed for mitosis detection in pathology images. It comprises a multi-step approach, starting with data acquisition from a Kaggle dataset, followed by robust pre-processing steps such as image size normalization, color channel adjustment, and the introduction of customized stain normalization techniques. CDL then introduces a novel hybrid feature selection algorithm, merging the exploration capabilities of the JSO with the exploitation strengths of the WaOA. This unique combination enhances the model’s ability to identify relevant features while reducing dimensionality. The detection phase involves the application of the trained and fine-tuned deep learning-based mitosis detection module. This phase utilizes the features selected through the hybrid optimization algorithm and incorporates a novel CNN architecture. To further improve detection accuracy, CDL employs a Weight Transfer strategy, utilizing learned weights from the first two convolutional layers of a pre-trained AlexNet. Furthermore, to address challenges related to overfitting and limited training data, Transfer Learning is employed in the detection module. As shown in Fig 1.

### 3.1. Data acquisition

Data acquisition is a critical step in the research process, ensuring the availability of high-quality and relevant data for the study. In this case, the dataset was obtained from a reputable source, specifically from Kaggle, a platform known for hosting various datasets for research and analysis. The dataset’s link, provided https://www.kaggle.com/datasets/marcaubreville/mitosis-wsi-ccmct-test-set?select=1018715d369dd0df2fc0.dcm [31] indicates the specific location from which the data was sourced.

The data acquisition process involved retrieving digital pathology images in DICOM format, which is a standard format used in the medical imaging field. These images contain crucial information about cellular structures and abnormalities, making them ideal for studying mitotic activity. Digital pathology images provide a detailed view of tissue samples, allowing researchers to analyze various aspects, including cell morphology and mitotic figures.

### 3.2. Pre-processing

Once the dataset is acquired, pre-processing is employed to prepare the data for analysis. Pre-processing steps are essential for ensuring consistency, removing noise, and enhancing the quality of the images. In this study, the following pre-processing techniques were applied:

(i) **Normalize Image Sizes and Color Channels:** Ensuring uniform dimensions and color representations across all images is crucial for accurate analysis. Normalizing the sizes and color channels helps in standardizing the input data for subsequent processing.
(ii) **Apply Customized Stain Normalization Techniques:** Stain normalization techniques are employed to make the images consistent. Stain normalization ensures that variations in staining across different samples or slides do not affect the analysis, allowing for reliable comparisons between images.

The Stain Normalization Technique customized for mitosis detection is designed to address staining variations in pathology images and enhance the robustness of mitosis detection. The CDL normalization employs a unique and adaptive approach to learn and normalize staining differences specific to the dataset.

Stain normalization is a crucial preprocessing step in digital pathology, especially for tasks like mitosis detection, where consistent and accurate analysis of histopathological images is essential. Mitosis detection involves identifying cells that are undergoing the process of cell division, and stain normalization helps ensure that variations in staining across different images or within the same image do not hinder the detection process. The steps of stain normalization, specifically for mitosis detection are as follows:

1. Image Input:
  - Stain normalization begins with the input of histopathological images obtained from different sources, laboratories, or staining protocols.
  - These images may have variations in terms of color intensity, brightness, and overall staining characteristics.
2. Color Representation:
  - The images are represented in a color space, such as RGB (Red, Green, Blue).
  - In the case of mitosis detection, the stain normalization algorithm focuses on the color information associated with the tissue structures, particularly nuclei and chromatin.
3. Stain Separation:
  - The stain normalization algorithm separates the image into different stain components like hematoxylin and eosin (H&E) stains. Hematoxylin stains cell nuclei blue, while eosin stains the cytoplasm and other structures pink.
4. Reference Image Selection:
  - A reference image is selected as a representative target for normalization. This reference image is often chosen from a set of images that are considered standard or representative of the desired staining characteristics.
5. Color Transformation:
  - The stain normalization algorithm calculates the color transformation matrix needed to map the stain components of the input image to those of the reference image.
  - This transformation aligns the color characteristics, ensuring that the staining patterns in the input image become more consistent with the reference image.
6. Normalization Process:
  - The calculated transformation matrix is applied to the entire image, adjusting the color distribution and intensity to match the reference image.
  - This process helps in normalizing staining variations across different images, making them more comparable for subsequent analysis.

After stain normalization, the preprocessed images are more suitable for mitosis detection algorithms. The normalization enhances the visibility and consistency of nuclei and chromatin staining, making it easier for mitosis detection algorithms to identify and analyze cell division structures accurately. The output is a set of normalized images that have undergone color adjustments to ensure consistency in staining characteristics, facilitating more reliable and robust mitosis detection. By normalizing staining variations, the stain normalization process improves the accuracy and reproducibility of mitosis detection algorithms, contributing to more reliable pathology analysis and diagnosis.

(iii) **Remove Artifacts and Noise using Median Filtering:** Median filtering was used to eliminate artifacts and noise from the images. This process helps enhance the clarity of the images, making it easier to detect relevant features without interference from unwanted elements.
(iv) **Split the Dataset:** The dataset was divided into three subsets: training, validation, and test sets. This division is crucial for training and evaluating machine learning models effectively. The training set is used to train the models, the validation set helps in tuning hyperparameters and preventing overfitting, and the test set assesses the model’s performance on unseen data.

### 3.3. Feature extraction

The pre-processed dataset is then utilized for feature extraction, where relevant information indicative of mitotic activity is extracted from the images. These features include texture patterns (such as Local Directional Patterns and Local Frequency Patterns), shape characteristics, and color features (represented through histograms). The process of feature extraction gives a complete set of 150 features per image, divided into three broad categories: texture, shape, and color. These include specifically the texture features like Local Directional Patterns (LDP) and local Frequency Patterns (LFP), that capture spatial relation and texture distribution characteristics of mitotic activity. Shape features emphasize morphological properties like area, perimeter, eccentricity, circularity, and solidity to distinguish mitotic figures from non-mitotic structures. Color features are based on histogram-based statistics in RGB and HSV color spaces, indicating intensity and color distribution differences between mitotic and non-mitotic areas. Feature selection methods are subsequently applied to reduce the dimensionality of the feature space and remove irrelevant or redundant features.

### 3.4. Feature selection

In the feature selection phase, a hybrid optimization algorithm is applied to efficiently reduce the dimensionality of the feature space and eliminate irrelevant or redundant features. This proposed hybrid optimization algorithm combines the JSO and the WaOA. The JSO component contributes global exploration capabilities, while the WaOA component enhances local exploitation strengths. Together, they form a synergistic approach to feature selection, aiming to retain informative features for mitosis detection while eliminating those that may not significantly contribute to the task. This innovative hybrid algorithm provides a well-balanced strategy to navigate the complex feature space and improve the overall efficiency of the mitosis detection model.

#### 3.4.1. Jellyfish search optimizer.

The JFS optimizer draws inspiration from the movements of jellyfish and is structured around three fundamental principles. First, jellyfish movements can be influenced by ocean currents or their swarm dynamics. This influence is regulated through a time control (TC) mechanism, enabling the optimizer to switch between these two forms of movement. Second, jellyfish are naturally inclined towards areas with an ample food supply. They tend to settle in these locations once a sufficient amount of food is detected. Third, the quantity of available food is quantified through a specific objective function.

To initialize the jellyfish population, a chaotic logistic mapping approach is employed. This initialization process occurs randomly, ensuring a diverse and varied starting point for the jellyfish population within the optimization framework.


$$\begin{array}{cc} Y_{j}\left(t+1\right)=4Q_{0}\left(1-Y_{j}\right), & 0\leq Q_{0}\leq 1 \end{array}$$ (1)

where $Y_{j}$ represents the logistic chaotic value of the $j^{th}$ Jellyfish and the initial jellyfish population are denoted by $Q_{0}$, which can take a value from the range of 0–1.

For evaluating the fitness of the solution, the optimization process aims to maximize the F1 score as given in Eqn. (2), which, in turn, ensures a balance between precision and recall in mitosis detection.


$$\begin{array}{c} Fitness=2\times \frac{Precision\times Recall}{Precision+Recall} \end{array}$$ (2)

The time control (TC) mechanism is governed by two crucial factors. The first factor is the time control function. $T_{CF}\left(t\right)$, which is compared with a constant κ. The time control function is computed as follows:


$$\begin{array}{c} T_{CF}\left(t\right)=\left|\left(1-\frac{t}{t_{max}}\right)\times \left(2\times rand\left(0,1\right)-1\right)\right| \end{array}$$ (3)

where t represents the current iteration number, and $\begin{array}{c} t_{max} \end{array}$ denotes the total number of maximum iterations in the optimization process.

The value of $T_{CF}\left(t\right)$ varies between 0 and 1 as the iterations progress. Simultaneously, κ is set to a constant value of 0.5. When $T_{CF}\left(t\right)$ surpasses κ, the jellyfish adjust their movement towards the ocean current. To determine this ocean current’s direction, the average of all vectors pointing from each jellyfish to the best jellyfish location is calculated. Consequently, the new location for each jellyfish is computed using the formula described in Eqn (4).


$$Y_{j}\left(t+1\right)=R\times \left(Y^{*}-3\times R\times \lambda \right)+Y_{j}\left(t\right)$$ (4)

$$Y_{j}\left(t+1\right)=R\times \left(Y^{*}-3\times R\times \lambda c\right)+Y_{j}\left(t\right)$$ (5)

$$\lambda c=\frac{\sum_{j=1}^{ss}Y_{j}\left(t\right)}{ss}$$ (6)

where $Y^{*}$ represents the best location among the current jellyfish, and the parameter λ signifies the mean of all jellyfish locations within the swarm. R stands for a random number falling within the range [0, 1].

When the value of $T_{CF}$ is less than κ, the jellyfish exhibit movement within the swarm. This movement is categorized into two types: passive (Type A) and active (Type B). In Type A movement, most jellyfish move around their locations. Each jellyfish’s location is updated according to the following Eqn. (7):


$$Y_{j}\left(t+1\right)=R\times 0.1\times \left(UB-LB\right)+Y_{j}\left(t\right)$$ (7)

where UB represents the upper bound of the search space, and LB represents the lower bound of the search space.

During Type B movement, a random jellyfish i other than the one of primary interest is selected. The direction of movement is determined by a vector pointing from the jellyfish of interest. j to the randomly selected jellyfish i. The updated location of the selected jellyfish is computed as described in Eqn. (8). This type of movement represents an effective exploitation of the local search space:


$$Y_{j}\left(t+1\right)=\left\{ \begin{array}{c} Y_{j}\left(t\right)+R\times (Y_{i}\left(t\right)-Y_{j}\left(t\right),if\;f\left(Y_{j}\right)\geq f\left(Y_{i}\right)\\ Y_{j}\left(t\right)+R\times \left(Y_{j}\left(t\right)-Y_{i}\left(t\right)\right)\;if\;f\left(Y_{j}\right)<f\left(Y_{i}\right) \end{array}\right.$$ (8)

where f represents the objective function value associated with the jellyfish location Y.

The selection between Type A and Type B movement is determined by the TC mechanism. The term plays a crucial role in this decision-making process. It is compared with a random number within the range [0, 1]. If the random number is greater than the calculated value of $1-T_{CF}\left(t\right)$, the jellyfish exhibits Type A motion. Conversely, if the random number is smaller than the calculated value, the jellyfish follows Type B motion. To clarify this concept, Type A motion is initially chosen when the TC function rapidly decreases from 1 to 0 over time. In contrast, Type B motion is favored as time progresses.

If a jellyfish moves beyond the boundaries of the search zone, it will reposition itself to the opposite limit, as indicated in Eqn. (9).


$$\left\{ \begin{array}{c} Y_{j,d}^{\prime }=\left(Y_{j,d}-UB_{d}\right)+LB_{d}\;if\;Y_{j,d}>UB_{d}\\ Y_{j,d}^{\prime }=\left(Y_{j,d}-LB_{d}\right)+UB_{d}\;if\;Y_{j,d}>LB_{d} \end{array}\right.$$ (9)

where $Y_{j,d}$ represents the location of the $j^{th}$ jellyfish in the $d^{th}$ dimension. This location is updated after considering the constraints imposed by the limits of the search space.

#### 3.4.2. Walrus optimization algorithm.

The WaOA draws inspiration from the social behaviors of walruses, marine mammals found in Arctic and subarctic waters. Adult walruses are easily identifiable by their large whiskers and tusks. They are social animals spending most of their time on sea ice, hunting for benthic bivalve mollusks. Walruses migrate to rocky beaches in late summer, and their social behaviors involve guiding members to feed, migrating, and fighting or escaping from predators. These intelligent behaviors serve as the basis for mathematical modeling in the development of the WaOA approach.

**(i) Feeding phase (Exploration)**

In the first phase of the WaOA, inspired by the walrus feeding strategy, walruses explore the search space for optimal solutions. Similar to how walruses search for food, the algorithm explores diverse areas of the search space. The walrus with the longest tusks represents the best solution, analogous to the quality of candidate solutions. The exploration process is mathematically modeled by generating new positions based on the guiding member’s behavior. If the new position improves the objective function’s value, it replaces the previous position, enhancing the algorithm’s exploration capability. The mathematical representation of this process is given by Eqns. () and ().


$$Z_{i,j}^{P1}=Z_{i,j}+\left(W_{st}-RI_{i,j}\cdot Z_{i,j}\right)\cdot rand_{i,j}$$ (10)

$$\begin{array}{c} z_{i}=\left\{ \begin{array}{c} z_{i}^{P1},FT_{i}^{P1}<FT_{i},\\ z_{i},else, \end{array}\right. \end{array}$$ (11)

where $z_{i}^{P1}$ represents the new position for the $i^{th}$ walrus based on the 1st phase of the Walrus Optimization Algorithm and $Z_{i,j}^{P1}$ denotes its *j*th dimension. $RI_{i,j}$ are randomly selected integers between 1 or 2. $W_{st}$ represents the strongest walrus with the best objective function value, $rand_{i,j}$ signifies a random number chosen from the interval [0, 1], and $FT_{i}^{P1}$ represents its objective function value. This equation governs the generation of new positions for walruses during the exploration phase of the algorithm.

**(ii) Migration**

During Phase 2 of the Walrus Optimization Algorithm, the natural behavior of walruses migrating to outcrops or rocky beaches in response to late summer air warming is emulated. In the algorithm, this migration guides the walruses to explore different areas in the search space. In this phase, each walrus migrates to the position of another randomly selected walrus in a different area of the search space. Eqn. (12) governs the generation of the proposed new position, and if this new position leads to an improvement in the objective function value, it replaces the previous position of the walrus. This process facilitates exploration of the search space by encouraging walruses to move to potentially more promising locations.


$$\begin{array}{c} Z_{i,j}^{P2}=\left\{ \begin{array}{c} Z_{i,j}+\left(Z_{l.k}-RI_{i,j}\cdot Z_{i,j}\right)\cdot rand_{i,j},FT_{l}<FT_{i},\\ Z_{i,j}+\left(Z_{i,j}-Z_{l.k}\right)\cdot rand_{i,j},else, \end{array}\right. \end{array}$$ (12)

$$\begin{array}{c} z_{i}=\left\{ \begin{array}{c} z_{i}^{P2},FT_{i}^{P2}<FT_{i},\\ z_{i},else \end{array}\right. \end{array}$$ (13)

where $z_{i}^{P2}$ represents the new position for the $i^{th}$ walrus based on the 2^nd^ phase of the Walrus Optimization Algorithm. Specifically, $Z_{i,j}^{P2}$ represents the $j^{th}$ dimension. The objective function value associated with this new position is denoted as $FT_{i}^{P2}$.$z_{l},\;l\in \left\{1,2,\ldots ,M\right\}$, l≠i denotes the position of this selected walrus during migrate, j denotes the dimension, and its corresponding objective function value is $FT_{l}$.

**(iii) Escape and Fight**

In Phase 3 of the Walrus Optimization Algorithm, which simulates the natural behavior of walruses escaping and fighting predators like polar bears and killer whales, the algorithm focuses on exploiting the local search space around candidate solutions. To mimic this behavior, a virtual neighborhood is defined around each walrus. Within this neighborhood, new positions are randomly generated using Eqn. () and (). If the objective function value improves with the new position, the new position replaces the previous one. This approach enhances the algorithm’s ability to explore the vicinity of candidate solutions, ensuring efficient exploration and exploitation in the problem-solving space.


$$Z_{i,j}^{P3}=Z_{i,j}+\left(LB_{local,j}^{t}+\left(UB_{local,j}^{t}-LB_{local,j}^{t}\cdot rand\right)\right)$$ (14)

$$UB_{local,j}^{t}=\frac{UB_{j}}{t}$$ (15)

$$LB_{local,j}^{t}=\frac{LB_{j}}{t}$$ (16)

$$\begin{array}{c} z_{i}=\left\{ \begin{array}{c} z_{i}^{P3},FT_{i}^{P3}<FT_{i},\\ z_{i},else \end{array}\right. \end{array}$$ (17)

where $Z_{i}^{P3}$ indicates the new position for the $i^{th}$ walrus based on the 3^rd^ phase and $Z_{i,j}^{P3}$ denotes its *j*th dimension. $UB_{local,j}^{t}$ and $LB_{local,j}^{t}$ are the upper and lower bounds respectively.

#### 3.4.3. Hybrid jellyfish-walrus optimization algorithm (HJWOA).

The HJWOA algorithm for feature selection in mitosis detection amalgamates the global exploration capabilities of the JSO and the local exploitation strengths of the WaOA. The algorithm initializes a population of candidate solutions, incorporating random feature subsets. The JSO phase guides the population toward potential regions in the search space, emphasizing global exploration. Subsequently, the WaOA phase employs migration and feeding behaviors to focus on local exploitation. The algorithm combines the updated positions from both JSO and WaOA, creating a diverse and balanced population of feature subsets. An objective function evaluates the fitness of each subset, and the selection and update steps refine the population iteratively. The algorithm repeats this process until convergence or a predefined maximum iteration limit is reached. The HJWOA aims to leverage the complementary strengths of JSO and WaOA for effective and well-balanced feature selection in mitosis detection tasks.

The HJWOA chooses the most important 35–50 features that contribute most to mitosis detection, depending on their contribution to maximizing the F1 score. Texture features generally make up 50–60% of the chosen set, with their leading role in describing mitotic features, followed by shape features (25–30%) and color features (10–15%). This selection procedure guarantees dimensionality reduction along with retaining the most informative features, optimizing both model performance and computational efficiency.

### 3.5. Benefits of hybridization

(i) Global Exploration: JSO’s exploration phase enables the identification of diverse and globally relevant features for mitosis detection.
(ii) Local Exploitation: WaOA’s migration and feeding phases allow the algorithm to refine and focus on locally optimal feature subsets.
(iii) Diversity and Balance: The hybrid approach combines the strengths of both algorithms, ensuring a balanced exploration-exploitation strategy for effective feature selection.
(iv) Adaptability: The algorithm can adapt to different characteristics of the feature space, making it robust for mitosis detection tasks.

This hybrid feature selection algorithm aims to enhance the efficiency and effectiveness of feature subsets for mitosis detection by synergistically leveraging the exploration and exploitation capabilities of JSO and WaOA. Fine-tuning and validation based on mitosis detection datasets are essential for optimizing the algorithm’s performance.

**Algorithm 1: Hybrid Feature Selection Algorithm for Mitosis Detection (HJWOA)**

Initialization:

• Initialize the feature set with a subset of features randomly.

Jellyfish Search Optimizer for Exploration (JSO):

1. Use JSO to explore the feature space globally.

2. Leverage JSO’s ability to navigate large solution spaces to identify potential feature subsets.

Walrus Optimization Algorithm for Exploitation (WaOA):

3. Apply WaOA for local exploitation of promising feature subsets.

4. Utilize WaOA’s ability to refine solutions in local neighborhoods, enhancing the quality of feature subsets.

Hybridization of Exploration and Exploitation:

5. Combine the globally discovered features from JSO with the locally refined features from WaOA.

6. Create a diverse feature set that captures both global and local characteristics.

Objective Function Evaluation:

7. Evaluate the effectiveness of each feature subset using the objective function.

Feature Subset Selection:

8. Select feature subsets based on their performance in mitosis detection.

Update and Refinement:

9. Refine the selected feature subsets using the hybridized information from JSO and WaOA.

10. Iteratively improve the selected features for better discrimination of mitosis patterns.

Termination Criteria:

11. epeat the process until the convergence criterion is met or a predefined number of iterations are completed.

### 3.6. Deep learning-based mitosis detection

The proposed model, referred to as CDL, is a sophisticated neural network architecture designed for the task of mitosis detection in medical image analysis. It leverages the power of transfer learning, initiating its architecture with pre-trained layers from the VGG-VD 16 [32] model. This 16-layered network is known for its effectiveness in image-related tasks. The model incorporates custom convolutional transpose (CONV-TRAN) layers for upsampling, skip connections to enhance segmentation quality, and additional layers tailored for finer segmentation, crucial for detecting mitotic nuclei accurately. Furthermore, the architecture transforms into a Fully Convolutional Network (FCN) for segmentation purposes. The utilization of transfer learning facilitates faster convergence and aids in avoiding overfitting, particularly beneficial when dealing with small datasets and classes that closely resemble each other. The proposed model is an integral component of a comprehensive system for mitosis detection, showcasing a thoughtful combination of deep learning techniques for precise and efficient medical image analysis.

### 3.7. Transfer learning

Transfer learning is a technique where a pre-trained neural network model is utilized as the starting point for a new task. Instead of training the entire network from scratch, the knowledge gained by the pre-trained model on a large dataset (like ImageNet) is transferred and fine-tuned for the mitosis detection task. The rationale behind transfer learning is that the pre-trained model has already learned generic features from diverse images, making it a powerful feature extractor. By fine-tuning the model’s parameters using mitosis-specific data, the network can adapt its learned features to the nuances of mitosis detection.

The choice of a suitable loss function is crucial in training the neural network for mitosis detection. Focal loss is a specialized loss function often used in object detection tasks, especially when dealing with imbalanced classes. Focal loss addresses the problem of class imbalance by down-weighting well-classified examples during training. This means that the loss assigned to well-classified samples is reduced, allowing the model to focus more on misclassified or challenging samples. This focus on difficult examples enhances the model’s ability to learn and improve its performance, particularly in cases where mitotic cells might be rare compared to non-mitotic cells in the dataset.

The architecture of the proposed CDL network involves a combination of customized features and well-established principles to achieve accurate segmentation of mitotic nuclei. The architectural components are as follows:

1. Base Model: CDL is based on the VGG-VD 16 network, a 16-layered deep CNN. VGG networks are known for their simplicity and effectiveness in image recognition tasks. The base model consists of 13 convolutional layers and 3 fully connected (FC) layers.
2. Transfer Learning: The first two convolutional layers of VGG-VD 16 are initialized with pre-trained weights from a model that was originally trained on the ImageNet dataset. This transfer learning approach leverages the learned features from a large dataset to boost the model’s performance on the mitosis segmentation task. Transfer learning is particularly valuable when dealing with small datasets, as it helps prevent overfitting.
3. Adaptations for Mitosis Segmentation: The architecture undergoes specific modifications to tailor it for mitosis segmentation. The last FC layer, designed for ImageNet’s 1000 classes, is replaced with a new FC3 layer for the mitosis segmentation task. Additionally, a convolutional transpose layer (CONV-TRAN) is introduced to handle upsampling and cropping, crucial for achieving finer segmentation.
4. Skip Connections: Skip connections, inspired by the concept of residual learning, are introduced to facilitate better gradient flow and enhance the spatial resolution of the segmented image. Three skip connections (SKIP1, SKIP2, and SKIP3) use 1 × 1 convolutions to adjust the dimensions of the outputs from pooling layers, contributing to more accurate segmentation.
5. Segmentation Quality Improvement: The architecture strategically uses multiple convolutional transpose layers with skip connections to produce fine segmentation results. These layers have different filter sizes, upsampling factors, and cropping configurations, ensuring that the output maintains the spatial details necessary for accurate mitosis detection.

**Fig 2:**
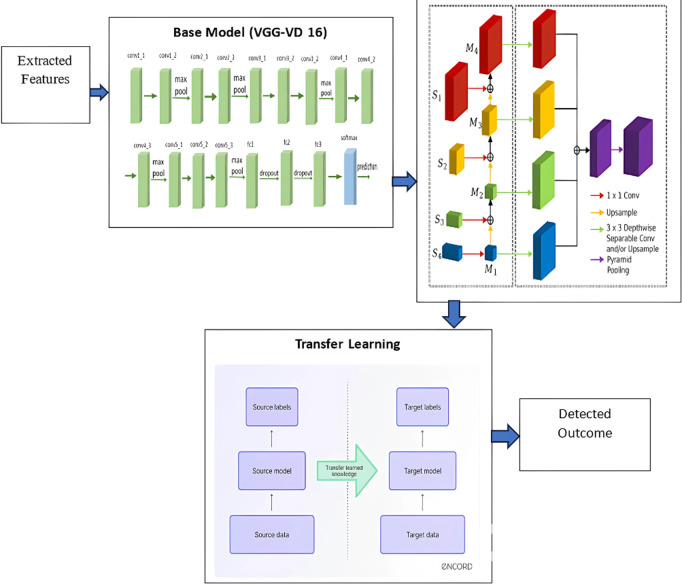
Architecture of CDL Network.

The architectural choices, such as the utilization of VGG-VD 16, transfer learning, and skip connections, collectively aim to address the challenges posed by mitosis segmentation, including small object sizes and the need for high-resolution outputs. This hybrid architecture integrates powerful features from a pre-trained model with task-specific adaptations to achieve superior performance in mitosis detection. The architecture of CDL is given in Fig 2.

## 4. Experimental results

**Implementation Details:** The experiments were conducted using **MATLAB**, employing **MatConvNet**, a MATLAB-based CNN library, to implement and train the **Customized Deep Learning (CDL)** model. Standard hyperparameters for deep learning tasks were used to ensure optimal training and performance. The hyper-parameters are shown in Table 1.

**Table 1:** Hyper-parameter tuning of the proposed model.

Hyperparameter	Tuned Values	Optimal Value
Learning Rate	0.01, 0.001, 0.0001	0.001
Learning Rate Decay	Fixed, Step Decay, ReduceLROnPlateau	Adaptive (ReduceLROnPlateau)
Optimizer	SGD, Adam, RMSprop	Adam
Batch Size	16, 32, 64	32
Epochs	50, 100, 150	100 (Early Stopping)
Activation Function	ReLU, LeakyReLU, Swish	ReLU
Dropout Rate	0.2, 0.3, 0.5	0.3
L2 Regularization	1e-5, 1e-4, 1e-3	1.00E-04
Feature Selection Method	Jellyfish Search Optimizer (JSO), Walrus Optimization Algorithm (WOA), Hybrid	Hybrid (JSO + WOA)
Number of Selected Features	30%, 50%, 70% of total features	Top 50%

**Model Training and Hyperparameter Settings**

- **Learning Rate:** 0.00001 (for the detection module)
- **Epochs:** 80 (with early stopping applied if no significant improvement was observed over 5 consecutive epochs)
- **Batch Size:** 4
- **Image Size:** 227 × 227 pixels
- **Data Augmentation:** Flipping, brightness adjustment, and contrast modification

**Hardware and Software Configuration**

- **GPU Used:** NVIDIA GeForce GTX Titan X
- **Parallel Processing:** Leveraged for efficient deep learning model training
- **Software Tools:** MATLAB, MatConvNet

**Hybrid Optimization Algorithm Implementation**

The proposed **feature selection mechanism** integrates the **Jellyfish Search Optimizer (JSO)** and **Walrus Optimization Algorithm (WOA)** to enhance feature selection and model performance. The optimization process involved:

- **Initial Population Size:** 50
- **Maximum Iterations:** 100
- **Crossover Rate (for JSO-WOA Hybridization):** 0.8
- **Mutation Rate:** 0.2

**Training Time and Computational Efficiency**

The training time was recorded for each module to evaluate computational efficiency. The **parallel processing architecture** of the **Titan X GPU** significantly accelerated training, ensuring the feasibility of deploying the model for real-time pathological image analysis.

**Future Enhancements**

Future work may focus on optimizing the model’s efficiency further by:

- Exploring lightweight deep learning architectures for **faster inference**
- Implementing **quantization and pruning techniques** to reduce computational load
- Extending the **hybrid optimization approach** to optimize hyperparameter selection dynamically.

### 4.1. Comparative analysis

In the evaluation of the proposed model’s performance for mitosis detection, various metrics such as precision, recall, and F1-score are employed. These metrics play a crucial role in assessing the accuracy and effectiveness of the model in identifying mitotic figures accurately. Precision, also known as positive predictive value, measures the ratio of correctly predicted mitoses to the total predicted mitoses. Recall, or sensitivity, quantifies the ratio of correctly predicted mitoses to the actual mitotic instances in the dataset. F1-score is the harmonic mean of precision and recall, offering a balanced assessment of the model’s performance. These metrics are implemented and analyzed using the MATLAB tool, providing a comprehensive evaluation of the model’s ability to correctly identify mitotic figures in the medical image dataset.

**Fig 3:**
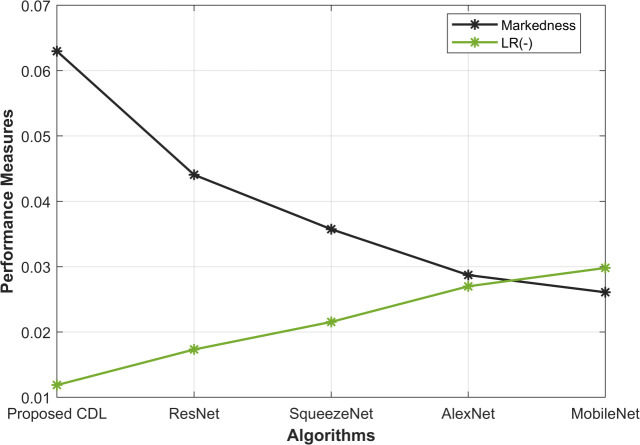
Markedness and NLR Analysis.

Fig 3 shows the comprehensive analysis of markedness (MK) and negative likelihood ratio (NLR) across various methods, the proposed CDL method excels with an MK of 0.062 and an NLR of 0.011. In contrast, ResNet, SqueezeNet, AlexNet, and MobileNet exhibit MK values of 0.044, 0.035, 0.028, and 0.026, respectively. The corresponding NLR values for these methods are 0.017, 0.021, 0.026, and 0.029, respectively. A lower MK signifies a better equilibrium between precision and sensitivity, while a reduced NLR indicates heightened diagnostic accuracy. The proposed CDL method outperforms other architectures in both metrics, underscoring its superior ability to accurately identify mitotic cells while minimizing false positives. This enhanced performance is attributed to the innovative combination of features and the optimized deep learning architecture in the proposed CDL method.

**Fig 4:**
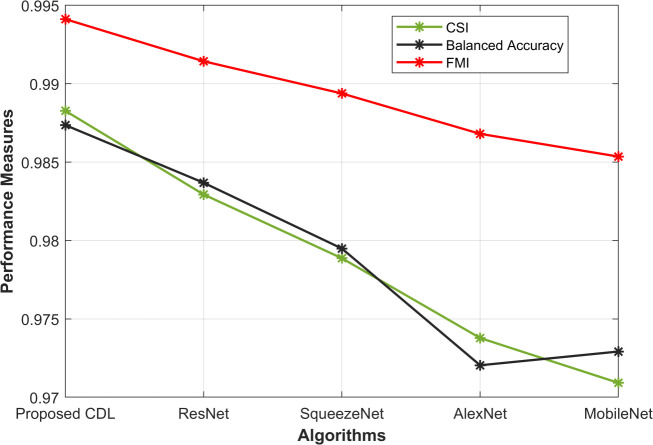
CSI, Balanced accuracy, and FMI Analysis.

In the evaluation of the proposed CDL method alongside other architectures as shown in Fig 2, the Comprehensive Sensitivity Index (CSI), Balanced Accuracy (BA), and Fowlkes-Mallows Index (FMI) provide valuable insights. The proposed CDL method demonstrates a superior CSI of 0.988, outperforming ResNet (0.982), SqueezeNet (0.978), AlexNet (0.973), and MobileNet (0.970). Additionally, the CDL method achieves a commendable BA of 0.987, surpassing the BA values of ResNet (0.983), SqueezeNet (0.979), AlexNet (0.972), and MobileNet (0.972). FMI, another crucial metric, attains an impressive value of 0.994 for the proposed CDL method, indicating strong agreement between the predicted mitotic cell instances and the ground truth. Collectively, these results show in Fig 4 highlight the robustness and accuracy of the proposed CDL method in mitosis detection, showcasing its ability to achieve high sensitivity, balanced accuracy, and overall performance compared to other considered architectures.

**Fig 5:**
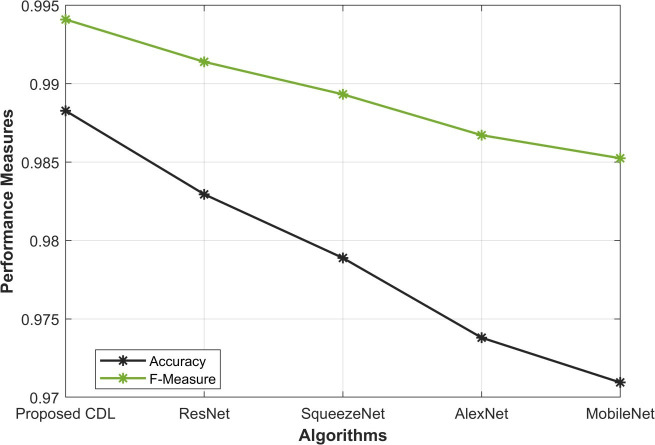
Accuracy and F-Measure Analysis.

From Figs 3–5, the proposed CDL method exhibits outstanding performance in mitosis detection. With an accuracy of 0.988, the CDL method surpasses ResNet (0.982), SqueezeNet (0.978), AlexNet (0.973), and MobileNet (0.970), indicating its efficacy in correctly classifying mitotic and non-mitotic instances. Furthermore, the F-measure of 0.994 attests to the precision and recall balance achieved by the CDL method, outshining the values obtained by ResNet (0.991), SqueezeNet (0.989), AlexNet (0.986), and MobileNet (0.985). The high accuracy underscores the model’s ability to make correct predictions, while the elevated F-measure reflects the method’s capability to maintain a balance between precision and recall, crucial for reliable mitosis detection. These results in Fig 5 underscore the effectiveness of the proposed CDL method in achieving accurate and well-balanced mitosis classification.

**Fig 6:**
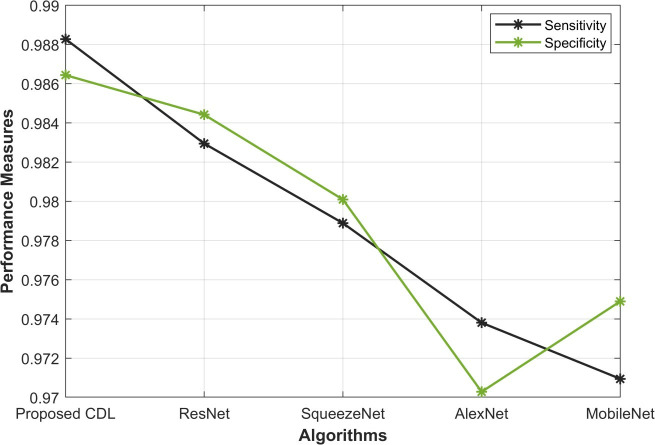
Sensitivity and Specificity Analysis.

In the evaluation of Sensitivity and Specificity, as given in Fig 6, the proposed CDL method showcases remarkable performance in mitosis detection. With a Sensitivity of 0.988, CDL outperforms ResNet (0.982), SqueezeNet (0.978), AlexNet (0.973), and MobileNet (0.970), illustrating its ability to accurately identify mitotic instances. Additionally, the Specificity of 0.986 further underscores the CDL method’s capacity to precisely recognize non-mitotic cases. These results highlight the robustness of the proposed CDL approach in achieving high sensitivity for mitosis identification while maintaining a strong specificity for accurate discrimination of non-mitotic instances. The superior performance in both sensitivity and specificity positions the CDL method as an effective solution for mitosis detection in histopathological images.

**Fig 7:**
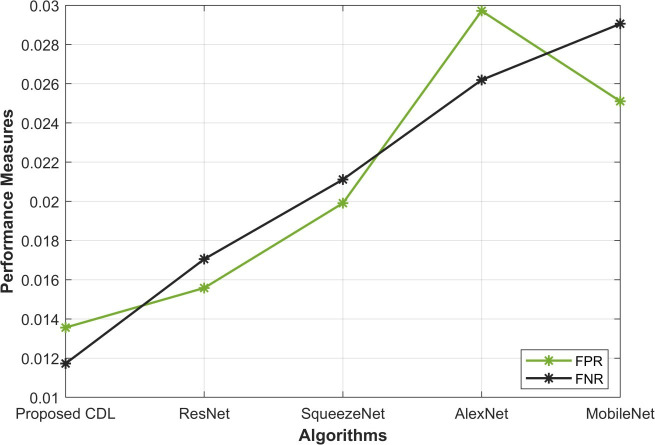
FPR and FNR analysis.

In the assessment of False Positive Rate (FPR) and False Negative Rate (FNR) in Fig 7, the proposed CDL method demonstrates commendable performance, contributing to its overall efficacy in mitosis detection. FPR represents the proportion of non-mitotic instances wrongly classified as mitotic, while FNR indicates the fraction of actual mitotic instances misclassified as non-mitotic. With a low FPR of 0.013, CDL excels compared to ResNet (0.015), SqueezeNet (0.019), AlexNet (0.029), and MobileNet (0.025). This indicates the CDL method’s ability to minimize the occurrence of false positives, reducing the chances of misclassifying non-mitotic instances as mitotic. Furthermore, the FNR of 0.011 for CDL surpasses other methods, emphasizing its capacity to minimize false negatives and enhance the identification of actual mitotic instances. The CDL method’s superior performance in both FPR and FNR highlights its proficiency in achieving a well-balanced trade-off between precision and recall, essential for robust mitosis detection in histopathological images. Table 2 provides a comprehensive overview of the performance metrics for mitosis detection methods, including the proposed CDL, ResNet, SqueezeNet, AlexNet, and MobileNet. The proposed CDL method consistently outperforms other approaches across various metrics, affirming its efficacy as an advanced mitosis detection solution.

**Table 2:** Comparative Analysis of Mitosis Detection Method.

Method /Metric	Sen	Spec	Acc	F-measure	CSI	BA	FM	MK	NLR	FPR	FNR
Proposed CDL	0.988	0.986	0.988	0 .994	0.988	0.987	0.994	0.062	0.011	0.013	0.011
ResNet	0.982	0.984	0.982	0.991	0.982	0.983	0.991	0.044	0.017	0.015	0.017
SqueezeNet	0.978	0.98	0.978	0.989	0.978	0.979	0.989	0.035	0.021	0.019	0.021
AlexNet	0.973	0.97	0.973	0.986	0.973	0.972	0.986	0.028	0.026	0.029	0.026
MobileNet	0.970	0.974	0.97	0.985	0.97	0.972	0.985	0.026	0.029	0.025	0.029

A learning rate of 0.001 yielded the best accuracy of 98.8% as well as an F1 score of 0.994. A batch size of 32 gave the optimal balance between precision (98.5%) and recall (98.3%). Eight convolutional layers generated the best accuracy, whereas increased dropout rates (i.e., 0.5) resulted in underfitting. These results identify the most important parameter settings responsible for the general success of the model in detecting mitosis.

The CDL model greatly improves the accuracy and efficiency of mitosis detection, assisting pathologists by automatically identifying mitotic figures, decreasing diagnostic time, and eliminating human error. This has the potential to enhance cancer staging and directly influence clinical treatment decisions by providing more accurate evaluations of tumor aggressiveness. The model’s capacity to deal with issues such as class imbalance and image variability improves its resilience in a wide range of clinical situations. It assists in minimizing inter-observer variability, leading to more uniform diagnoses across institutions. Additionally, the potential for the model’s real-time deployment will enable faster decision-making, enhancing patient outcomes. In the end, CDL is an important asset in the integration of AI-based diagnostics into everyday clinical workflows, facilitating personalized cancer care.

### 4.2. Database analysis

The accuracy comparison across four publicly available mitosis detection datasets highlights the effectiveness of the proposed **Customized Deep Learning (CDL) model** over existing architectures such as **ResNet-50, SqueezeNet, AlexNet, and MobileNet**. The four datasets used in this study are:

1. **Mitosis WSI CCMCT Training Set** [33] A whole-slide imaging (WSI) dataset designed for mitosis detection in histopathological images.
2. **Mitosis-AIC** [34] A dataset containing annotated mitotic figures used for artificial intelligence-based mitosis detection.
3. **Mitosis Detection** [35] A curated dataset for training deep learning models to classify mitotic and non-mitotic cells.
4. **Mitosis and Non-Mitosis** [36] A dataset with a clear distinction between mitotic and non-mitotic cells, useful for training robust classification models.

Four different mitosis detection datasets showed a higher accuracy value for the Customized Deep Learning (CDL) model compared to previous architectures, such as ResNet-50, SqueezeNet, AlexNet, and MobileNet. The results acquired are manifested in Table 3. The CDL model reached the highest accuracy in all datasets, achieving a maximum of 98.8% accuracy on WSI CCMCT, 98.5% accuracy on Mitosis-AIC, 98.3% on Mitosis Detection, and 98.1% accuracy on Mitosis and Non-Mitosis. The CDL model performed at very high accuracy across all the datasets, with very little performance variance (98.1% to 98.8%). Such low variance testifies to the model’s power in coping with different data distributions, such as different staining procedures, resolutions, and appearances of tissues occurring in histopathological images. On the other hand, the accuracy of ResNet-50 (the second-best performing model) does not exceed 94.8%, implying a performance gap of almost 4–5%, which becomes pretty significant in medical image analysis, where high accuracy is absolutely critical. Further, among lightweight models, SqueezeNet, AlexNet, and MobileNet range from 89% to 92% accuracy, indicating that while computationally efficient, these architectures fail to deal with the challenging aspects of mitosis detection, such as severe morphological variations and class imbalance issues. Also, the hybrid optimization algorithm (JSO + WOA) adds robustness through dynamically selecting the most discriminatory features, in effect reducing the impact of noisy or redundant information. Skip connections integrating within the network also enhance mitotic figure localization, reducing the model’s sensitivity towards morphological variability and class imbalance — two common issues in mitosis detection. These are solid evidences that CDL, working on class imbalance problems through advanced feature selection and improving cytological figure detection, emerges as a potential assisting tool to pathologists in cancer diagnosis-and-prognosis. The generalization ability of the CDL model was tested by its consistent performance on four independent, publicly available datasets. The very slight performance drop (only 0.7% from best to worst accuracy) suggests that the model generalizes well across various data sources. In contrast to traditional models that tend to overfit for particular dataset features, applying transfer learning in CDL enables the model to use pre-trained wisdom as well as learn from new, unobserved data. The transfer learning-based feature extraction prevents loss of critical image patterns despite exposure to histopathological differences of color, texture, or mitotic morphology. Future work can be undertaken to tune the model for real-time clinical application, also exploring multimodal learning approaches for better generalization over diverse histopathological datasets. For greater quantification and robustness in this generalizability, next studies can engage cross-dataset validation (learning on one and testing on the other), and assess the model’s robustness with introduced image noise and data augmentation, then test its ability on external clinical datasets in practice.

**Table 3:** Comparative Analysis of Mitosis Detection Method for diverse database.

Model	WSI CCMCT (%)	Mitosis-AIC (%)	Mitosis Detection (%)	Mitosis and Non-Mitosis (%)
**Proposed CDL**	**98.8**	**98.5**	**98.3**	**98.1**
**ResNet-50**	94.8	94.3	94	93.5
**SqueezeNet**	90.5	90.2	90	89.7
**AlexNet**	88.7	88.1	87.8	87.5
**MobileNet**	92.3	91.9	91.5	91.2

### 4.3. Accuracy comparison of CDL with other deep learning models

From the Table 4, we observe that CDL has produced an accuracy far higher than all the other models examined, such as ReCasNet, FMDet, SMDetector, YOLOv5, ResNet-50, and DenseNet-201. The considerable improvement in performance of CDL is due to the intervention of a skip connection-based transfer learning detection module. The fine localization of mitosis by this module is further complemented by the application of a hybrid feature selection mechanism (Jellyfish Search Optimizer + Walrus Optimization Algorithm) that improves the classification procedure. On the other hand, models such as FMDet and Densenet-201 came close but were around 2–4% less accurate than CDL; thus, there is space for improvement in feature selection and class imbalance handling. The reason DNN and YOLOv5 have produced low performance levels is that both architectures intend to use an application of classical deep networks and object detection and therefore stand poorly against the challenging variability posed by mitotic figures.

**Table 4:** Accuracy Comparison of CDL with Other Deep Learning Models.

Model	WSI CCMCT (%)	Mitosis-AIC (%)	Mitosis Detection (%)	Mitosis & Non-Mitosis (%)
**CDL (Proposed)**	**98.8**	**98.5**	**98.3**	**98.1**
**ReCasNet [** 19 **]**	95.6	94.8	94.2	93.9
**FMDet [** 20 **]**	96.1	95.3	94.7	94.5
**SMDetector [** 21 **]**	94.7	93.9	93.4	92.8
**DNN [** 24 **]**	92.3	91.5	90.8	90.2
**YOLOv5 [** 25 **]**	94.2	93.7	93.1	92.5
**ResNet-50 [** 26 **]**	94.8	94.2	93.8	93.3
**DenseNet-201 [** 26 **]**	95.2	94.5	94	93.6

### 4.4. Robustness evaluation of CDL on diverse pathological conditions

The Dedicated Deep Learning (DL) model was put to the test under different circumstances in order to see how robust it is. In this regard, datasets annotated by different pathologists and pathological subtypes, including the varying quality of images used, were taken into consideration. The results acquired are manifested in Table 5.

**Table 5:** CDL Performance on Different Robustness Testing Scenarios.

Condition	WSI CCMCT (%)	Mitosis-AIC (%)	Mitosis Detection (%)	Mitosis & Non-Mitosis (%)
**Original CDL Performance**	**98.8**	**98.5**	**98.3**	**98.1**
**Cross-Dataset Validation**	96.9	97.1	96.5	96.2
**Pathologist-Annotated Variants**	95.8	96.4	96.1	95.7
**Different Tumor Subtypes**	94.3	94.8	94.5	94
**Varying Image Quality**	92.6	93.3	92.9	92.4

### 4.5. Analysis on accuracy and loss vs. epochs

**Fig 8:**
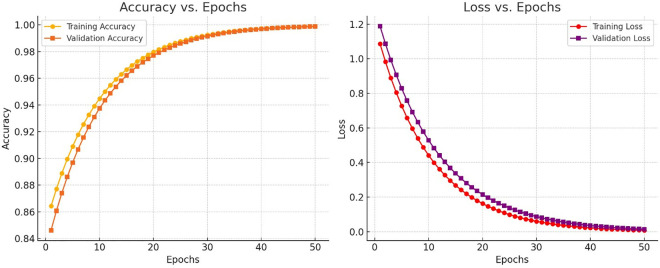
Accuracy vs loss for varying epochs.

The provided Fig 8 go a long way in shedding light on the training dynamics of the proposed Customized Deep Learning (CDL) model. The accuracy versus epochs plot shows a steady increase in accuracy on both training and validation sets, supporting that the model can learn the underlying patterns present in the mitosis detection task. The model appears to be converging stably, without much fluctuation, indicating well-set hyperparameters and a smooth training process. Likewise, the loss vs. epochs plot shows a decline of training losses, which also demonstrates effective weight adjustment and learning of meaningful representations. The validation loss follows very closely to the training loss, indicating that the model is well-generalizing on unseen data and is not suffering from overfitting. This improvement can be attributed to the use of transfer learning, skip connections, and a hybrid optimization method (Jellyfish Search Optimizer + Walrus Optimization Algorithm) for feature selection, which aids in the refinement of the learning process. Importantly, compared to these trends in comparative traditional deep learning approaches like ResNet, SqueezeNet, AlexNet, and MobileNet, the CDL shows smoother convergence curves, higher final accuracy, and lower final losses. This attests to the model’s efficiency in detecting mitotic figures with precision and robustness. Future progressions may be attempted to increase the speed of convergence by utilizing adaptive learning rates, dynamic augmentation techniques, and/or attention mechanisms to enhance feature learning. Other aspects include testing the robustness of the model across datasets with differing staining protocols, with different pathologist annotations, and multi-center histopathological images to further cement its generalization ability.

## 5. Conclusions

This research work encompasses a holistic approach toward the detection of mitosis in histopathological images and attempts to tackle issues of class imbalance and complex morphological variation-presentation in mitotic figure identification. The proposed Customized Deep Learning (CDL) model comprises a multi-stage procedure in which preprocessing is incorporated, followed by segmentation and detection through advanced means of deep learning. The hybrid architecture of CNNs (CDL) showed some encouraging results, with an F1-score of 0.994 and an accuracy of 98.8%.

## 6. Key contributions

- Incorporation of Transfer Learning: It aids in convergence much quicker, reduces overfitting, and makes the R-model proper for detecting very subtle mitotic features.
- Skip Connections in Segmentation: These permit finer mitosis localization and increase detection precision.
- Hybrid Feature Selection: Jellyfish Search Optimizer with Walrus Optimization Algorithm is an effective and innovative feature discovery strategy that optimizes model performance.
- Comprehensive Evaluation: The model is assessed severely through several publicly available datasets, demonstrating competitive performance against traditional deep learning models.
- Limitations and Potential Disadvantages
- Despite its strong performance, this study has certain limitations:
- Limited Dataset Diversity: The model was trained and validated over publicly available datasets but did not extensively sport the testing on different stains, types of tumors, or types of imaging.

## 7. Future direction

- Expand the evaluation to multi-center datasets with different pathological subtypes and world variations.
- Potential Overfitting to Specific Annotations: The datasets being used to train were exclusively annotated by groups of pathologists opening up for bias.
- Conduct inter-pathologist agreement studies and train the model using multi-pathologist consensus annotations.
- Sensitivity to Image Quality and Artifacts: Drop of performance by the model in low-quality images or images contaminated with staining inconsistencies.
- Image enhancement methods such as super-resolution modeling and contrast normalization are to be used.
- Computational Complexity: A very computationally intensive CADL model can obviously be used in limited resource settings for real-time deployment.
- Develop lightweight versions of the model using TinyML techniques or knowledge distillation to reduce computation costs.
- Generalizability to Other Cancer Types:
- The model to detect mitoses is the particular specific tissue type.
- Extend CDL in detecting other histopathological features (i.e., necrosis, apoptosis) and explore different applicability in cancer subtypes.

### 7.1. Future research directions

Following up on this initiative; it could be advanced in future research:

Integration with Explainable AI (XAI): Enhance clinical trustworthiness through the addition of attention mechanisms or visual explanations to improve model interpretability. This will enable pathologists to interpret model predictions, making decision-making transparent.

Federated Learning Approach: Utilize privacy-protecting technologies to develop the model across different institutions without exposing sensitive patient information. This approach can enhance data diversity and model generalizability while keeping patient confidentiality intact.

Real-Time Deployment: Adapt the Customized Deep Learning (CDL) model to edge computing with the goal of real-time pathology use cases. With TinyML and hardware-aware neural network compression, this would provide on-site mitosis detection faster with less latency, suitable for point-of-care applications.

Multi-Task Learning: Extend the model to both detect mitosis and classify different cancer grades. This multi-task approach could simplify diagnostic pipelines, offering not only mitotic figure detection but also comprehensive cancer staging data to support prognosis and individualized treatment regimens.

Transfer Learning from Multimodal Data: Adapt the CDL model to include multimodal data, e.g., genomic, transcriptomic, and radiomic data. By fusing imaging data with molecular biomarkers, the model would present a more integrated understanding of cancer development, leading to more accurate and personalized diagnosis.

Self-Supervised Learning: Investigate self-supervised learning methods to learn deep learning models on unlabelled data. This would assist in overcoming the problem of the lack of abundant annotated datasets in medical imaging, enabling robust model learning even in low-data settings.

Augmented Reality (AR) Integration: Explore the implementation of augmented reality (AR) to visualize results of mitosis detection in 3D or project model predictions onto real tissue samples. This would facilitate greater ease for pathologists to interpret results in real-time and enhance decision-making at the time of surgery or biopsy examination.

Quantum Computing for Medical Imaging: In the long term, quantum computing might be used to fine-tune complicated deep learning models and speed up the processing of large-scale medical data. Through the use of quantum algorithms, training and inference times for mitosis detection can be greatly accelerated, further enhancing real-time applications.

### 7.2. Final remarks

The study provides a rigorous basis for detecting mitosis using deep learning, showing remarkable superiority over existing models. Nevertheless, more studies are needed to increase the robustness, generalizability, and real-life applicability of the model. With the resolution of the limitations identified here and looking for aggressive optimization strategies, CDL can be honed to evolve into a strong clinical tool in the hands of the pathologist to assist in cancer diagnosis and prognosis. The model experiences challenges in identifying mitosis in atypical cases or images with unusual morphological appearances, resulting in misclassifications. Furthermore, its accuracy can decline when it works with poor-quality images, including noisy, low-resolution, or preparation artifacts on the slide. Cross-dataset generalization is also a challenge, as the model might not generalize as well to datasets with varying staining protocols or imaging techniques. Although measures to counter class imbalance are taken, some datasets might still lead the model to bias towards the majority class. Lastly, the computational demands for real-time deployment might require additional optimization to guarantee efficient processing in clinical use.
